# Respiration‐driven triboelectric nanogenerators for biomedical applications

**DOI:** 10.1002/eom2.12045

**Published:** 2020-08-09

**Authors:** Jun Li, Yin Long, Fan Yang, Xudong Wang

**Affiliations:** ^1^ Department of Materials Science and Engineering University of Wisconsin‐Madison Madison Wisconsin USA

**Keywords:** biomechanical energy harvesting, implantable medical devices, respiration, triboelectric nanogenerator, wearable medical devices

## Abstract

As a fundamental and ubiquitous body motion, respiration offers a large amount of biomechanical energy with an average power up to the Watt level through movements of multiple muscles. The energy from respiration featured with excellent stability, accessibility and continuality inspires the design and engineering of biomechanical energy harvesting devices, such as triboelectric nanogenerators (TENGs), to realize human‐powered electronics. This review article is thus dedicated to the emerging respiration‐driven TENG technology, covering fundamentals, applications, and perspectives. Specifically, the human breathing mechanics are first introduced serving as the base for the developments of TENG devices with different configurations. Biomedical applications including electrical energy generation, healthcare monitoring, air filtration, gas sensing, electrostimulation, and powering implantable medical devices are then analyzed focusing on the design‐application relationships. At last, current developments are summarized and critical challenges for driving these intriguing developments toward practical applications are discussed together with promising solutions.

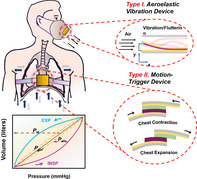

## Graphical abstract

Respiration is primarily an involuntary movement that consistently output biomechanical energy regardless of time and body status. It is considered as a continuous and stable body energy source that other voluntary body movements cannot compete with. This review article presents an overview of the major advancements in respiration‐driven triboelectric nanogenerators in the application area of wearable and implantable biomedical devices.

## 1 INTRODUCTION

Respiration is a fundamental and ubiquitous body movement that serves as a critical metabolic process of body exchanging oxygen and carbon dioxide. Besides, respiration is also a process of outputting work by converting chemical energy into mechanical energy, through the motion of intercostal muscles, lung muscles, and diaphragm. An average adult breathes at a normal rate of 12 to 16 times per minutes could provide a maximal power of up to the Watt level, which is a significant biomechanical energy source in human body and is adequate to support many small electronics.^1^, ^2^, ^3^, ^4^ If harvested effectively and appropriately, this energy source may allow us to bypass the bulky batteries in those electronics. More importantly, respiration is primarily an involuntary movement^5^, ^6^ (another typical one is heart beating) that consistently output biomechanical energy regardless of time and body status. It thus can be considered as a continuous and stable body energy source that other voluntary body movements (eg, muscle stretches from fingers, arms, legs, and feet^7^, ^8^) cannot compete with. Compared to heart beating, the motion from respiration is more accessible and has a higher energy density. Therefore, respiration could be the most appropriate biomechanical energy source for continuously powering wearable and implantable electronic devices to achieve 100% self‐powered battery‐free operation.

Among a number of mechanical energy harvesting principles, triboelectric nanogenerator (TENG) stands out with its simple configuration, light weight, large power density, high energy conversion efficiency, and durability.^9^, ^10^, ^11^, ^12^, ^13^, ^14^, ^15^ The last decade has witnessed big advancements of TENG from a basic concept^16^ to a well‐established technology holding promises in a wide range of applications.^17^, ^18^, ^19^, ^20^, ^21^, ^22^, ^23^, ^24^ The broad selection of biocompatible materials, particularly those with a high flexibility and stretchability, for TENG designs is an essential advantage for biomedical applications. For instance, TENGs has been applied as active sensors for real‐time healthcare monitoring through deciphering electrical signals into physiological information (eg, respiration,^25^, ^26^ heartbeats,^27^, ^28^ and blood pressure^29^). Implantable TENGs have been designed to generate μW‐level electricity from regular body movements, which was sufficient to power implantable medical devices (IMDs) such as pacemakers.^30^, ^31^ Intriguing interactions between TENG and cell/tissue/organ activities^32^, ^33^ were also discovered enabling self‐powered therapeutic stimulations.^34^, ^35^, ^36^, ^37^ Being aware of the unique advantages of biomechanical energy from respiration, a number of TENGs prototypes were designed and demonstrated, based on which intriguing biomedical applications were implemented. Although comprehensive review articles were published reporting the progress of TENG,^9^, ^10^, ^13^, ^38^, ^39^ there is no focused review dedicating to respiration‐driven TENG technology. To fill this gap, this article provides an overview of respiration‐driven TENG and its biomedical applications. The driving mechanics from respiration are first introduced serving as the base for designing TENG devices with different configurations. Applications including electrical energy generation, healthcare monitoring, air filtration, gas sensing, electrostimulation, and powering IMDs are then reviewed focusing on the design‐application relationships. At last, critical challenges for driving these intriguing developments toward practical applications are discussed together with potential solutions.

## 2 RESPIRATION‐DRIVEN TRIBOELECTRIC ENERGY HARVESTING

### 2.1 Respiration mechanics

**FIGURE 1:**
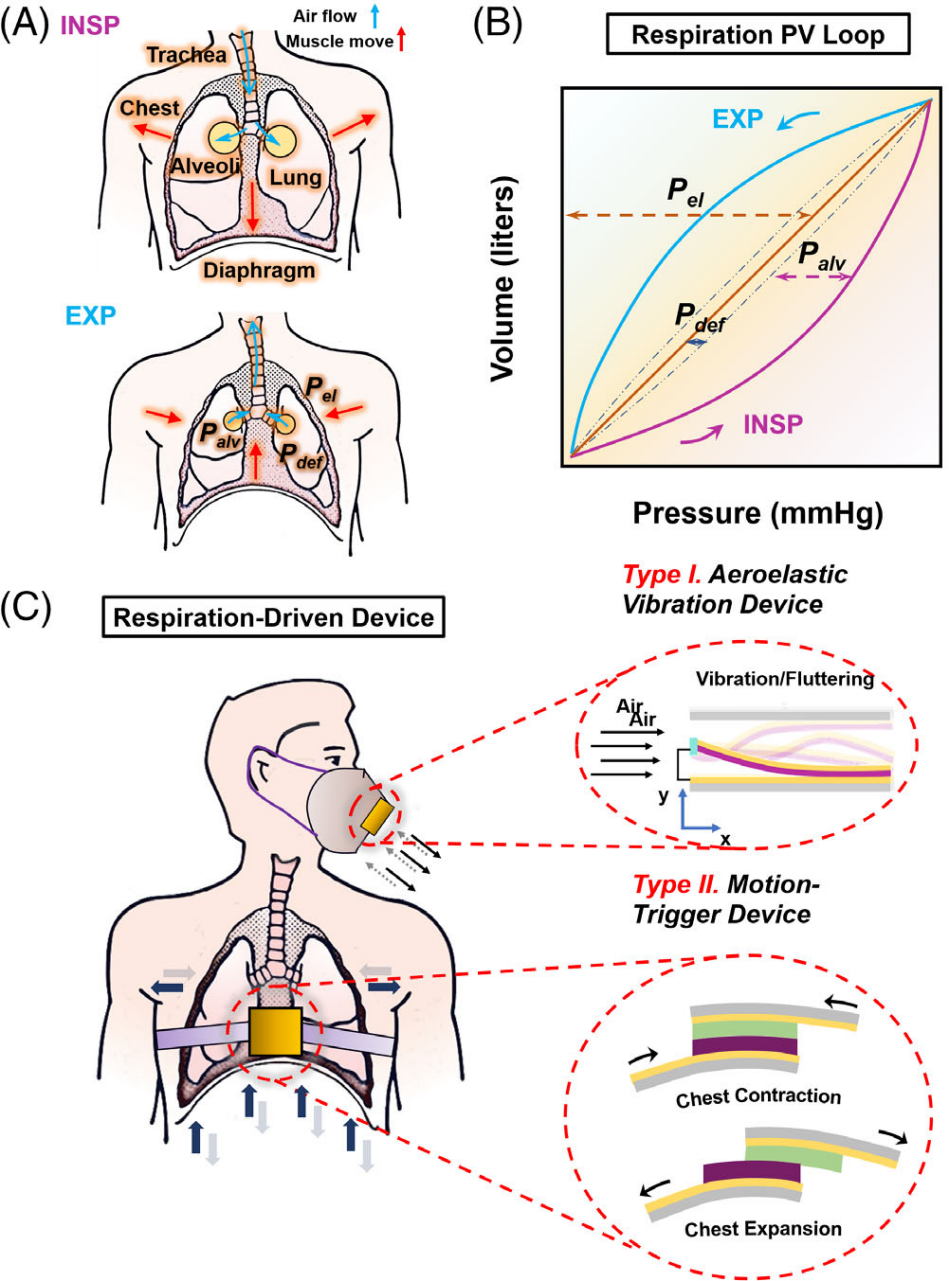
Mechanics of respiration. A, Schematics of muscle movement and air flow during inspiration (upper image) and expiration (bottom image). B, Typical pressure‐volume loop (one respiratory cycle) during breathing. *P*_el_, *P*_def_, and *P*_alv_ are represented by the abscissal distance from the axis of ordinates to the diagonal, from the diagonal to the dashed curve, and from the dashed curve to the loop, respectively. C, Schematics of two main types of respiration‐driven TENG that harvest energy from respiration at different locations. TENG, triboelectric nanogenerator

Breathing is a fundamental biological process that requires coordinated motions of different muscles with repeated constrictions and relaxations to realize the gas‐exchange process (Figure 1A). Specifically, during inhalation, both the diaphragm and intercostal muscles between ribs contract leading to chest expansion, which increases the intrapulmonary volume of lung and decreases the intrapulmonary pressure down by 1 to 2 mm Hg,^40^ forcing air in. In expiration, the relaxation of diaphragm and intercostal muscles decreases the intrapulmonary volume and increases pressure to 761 to 762 mm Hg, and thereby moves air out of lungs. In the case of heavy breathing, the movement of abdomen is also involved.

The mechanical energy associated with respiration‐related body motions has been well studied previously.^41^, ^42^, ^43^ To perform a respiration cycle, the body needs to overcome three major types of resisting forces, that is, elastic force of chest‐lung system (*P*_el_), air viscance and turbulence (*P*_alv_) due to pressure gradient between the alveoli and the mouth, and non‐elastic tissue deformation resistance (*P*_def_) (deformation of tissue between lung surface and the alveoli) (Figure 1B). Results based on empirical studies showed that among the overall mechanical energy consumed in respiration, 63% is to overcome the elastic force, 29% is related to air resistance, and the remaining 8% is to deform tissues. To approximate the total biomechanical energy from respiration, first, the linear elastic force could be expressed as:

(1) $$P_{\mathrm{el}}=\mathrm{KV},$$

where *K* is the elastance and *V* is the lung volume. Together, *P*_alv_ and *P*_def_ are estimated as following:

(2) $$P_{\mathrm{alv}}+P_{\mathrm{def}}=K_{1}\left(\frac{\mathrm{dV}}{\mathrm{dt}}\right)+K_{2}{\left(\frac{\mathrm{dV}}{\mathrm{dt}}\right)}^{2},$$

where (*dV/dt*) is the lung volume changing velocity and *K*_1_ and *K*_2_ are the constants. Therefore, the total force required for a respiration is:

(3) $$P_{\mathrm{tot}}=\mathrm{KV}+K_{1}\left(\frac{\mathrm{dV}}{\mathrm{dt}}\right)+K_{2}{\left(\frac{\mathrm{dV}}{\mathrm{dt}}\right)}^{2}.$$

Assuming that the velocity of volume change is a sine wave, which is close in a real scenario, the output mechanical work per respiration could be calculated as:

(4) $$W=\int P_{\mathrm{tot}}\mathrm{dV}=\frac{1}{2}{\mathrm{KV}}_{T}^{2}+\frac{1}{4}K_{1}\pi^{2}{\mathrm{fV}}_{T}^{2}+\frac{2}{3}K_{2}\;\pi^{2}f^{2}V_{T}^{3},$$

where *V*_T_ and *f* referring to the tidal volume and respiration frequency, respectively. Furthermore, expression of mechanical power could be deducted as

(5) $$P_{\text{power}}=\mathrm{Wf}=\frac{1}{2}{\mathrm{KfV}}_{T}^{2}+\frac{1}{4}K_{1}\pi^{2}{\left({\mathrm{fV}}_{T}\right)}^{2}+\frac{2}{3}K_{2}\;\pi^{2}{\left({\mathrm{fV}}_{T}\right)}^{3}.$$

According to Equation (5), given a reasonable respiration volume of 6 L at a frequency of 15/min, it can be estimated that respiration of an average adult in a resting status could generate a power around 80 mW.^41^, ^43^ For an air intake of 30 L/min under normal activity, the power value could reach to approximately 1 W.^1^ Impressively, heavy and rapid breath could even produce a mechanical power up to 44 W (at a frequency of 30/min and a maximal ventilation of 150 L/min that an individual could perform).^41^ In general, there is a considerable power availability from respiration if one takes account of the energy consumption of common electronics. Specifically, the sensor nodes have an power consumption at the *nW* or *μW* level.^44^, ^45^, ^46^ Most implantable medical devices require a power supply in the range of *μW* to *mW*.^47^, ^48^, ^49^ Even some portable electronics such as smart phone only demands 1 W of power.^50^ This comparison revealed the promises of using biomechanical energy from human respiration for powering most biomedical electronic devices.

### 2.2 TENG modes and fundamentals

All TENGs operate through the coupling of contact electrification and electrostatic induction.^10^, ^11^ Although TENG embodies a plethora of configurations and structures, there are four common operation modes in general, that is, vertical contact‐separation (CS), lateral in‐plane sliding, single‐electrode and freestanding mode, which have been comprehensively reviewed in a number of articles.^9^, ^10^, ^11^, ^13^, ^51^ Considering the specific motion patterns of respiration, to achieve high energy harvesting efficiency, most respiration‐driven TENGs were built based on the first two modes, that is, CS and sliding modes.

As generally known, the electricity in a CS mode TENG is generated by charge induction to balance the dynamic electrostatic field between triboelectric films that periodically separate and contact. The advantage of the CS mode is its high sensitivity to mechanical stimulus. However, the requirement of an air gap from separation raises engineering challenges for packaging and encapsulation. For a lateral sliding (LS) mode TENG, the electricity is generated by creating potential and electron flow between two electrodes through in‐plane displacing two triboelectric films back and forth. This mode is usually associated with enhanced output power compared to the CS mode especially when implementing a micrograting design; yet it requires larger area for operation. Numerically, for a CS mode TENG,^52^ the open‐circuit voltage (*V*_OC_) and short‐circuit current (*I*_SC_) can be expressed as:

(6) $$V_{\mathrm{OC}}=\frac{\mathrm{\sigma x}\left(t\right)}{\varepsilon_{0}},$$

(7) $$I_{\mathrm{SC}}=\frac{{\mathrm{dQ}}_{\mathrm{SC}}}{\mathrm{dt}}=\frac{\mathrm{I\omega \sigma}d_{0}}{{\left(d_{0}+x\left(t\right)\right)}^{2}}\frac{\mathrm{dx}}{\mathrm{dt}}=\frac{\mathrm{I\omega \sigma}d_{0}v\left(t\right)}{{\left(d_{0}+x\left(t\right)\right)}^{2}},$$

where *l* is the length, *w* is the width, *σ* is the charge density, *d*_0_ = *d*_1_/*ε*_1_ + *d*_2_/ *ε*_2_, *d*_1_ and *d*_2_ are the thickness, *ε*_1_ and *ε*_2_ are dielectric constants of dielectric films 1 and 2, and velocity *v*(*t*) = *dx*/*dt*, respectively. Analogously, $V_{\mathrm{OC}}^{\prime }$ and $I_{\mathrm{SC}}^{\prime }$ in the sliding mode^53^ can be expressed as a function of lateral displacement *x*(*t*) as:

(8) $$V_{\mathrm{OC}}^{\prime }=\frac{\mathrm{\sigma x}\left(t\right)d_{0}}{\varepsilon_{0}\left(1-x\left(t\right)\right)},$$

(9) $$I_{\mathrm{SC}}^{\prime }=\frac{dQ_{\mathrm{SC}}^{\prime }}{\mathrm{dt}}=\sigma \omega \frac{\mathrm{dx}}{\mathrm{dt}}=\mathrm{\sigma \omega v}\left(t\right).$$

Based on these equations, one could find that both modes have *V*_OC_ and *I*_SC_ related to variables due to mechanical stimuli (displacement or velocity of displacement). This relationship explains why simple deciphering the electricity signals of TENG could obtain essential physiological information such as respiration rate (associated with *v*(*t*)) and respiration depth (associated with *x*(*t*)).

### 2.3 Typical types of TENG for respiration energy harvesting

As mentioned in previous section, the chest movement and air flow contribute to the majority of biomechanical energy from respiration at 63% and 29%, respectively.^41^, ^42^ Therefore, two types of TENG devices have been designed in response to this two types of mechanical energy (Figure 1C). One type TENG is an aeroelastic vibration device activated by air flow during breathing. This device is capable of being integrated with face masks that usually contain a triboelectric beam with either a cantilever (one‐side fixed) or a bridge (two‐side fixed) configuration. It works based on the effect of aeroelastic flutter due to the coupling of aerodynamic force and elastic deformation.^54^, ^55^, ^56^, ^57^, ^58^, ^59^ Specifically, when the energy of air flow exceeds the mechanical damping of the triboelectric beam, beam vibration could be sustained with a large amplitude that drives the beam to touch the package case, resulting in triboelectrification between the beam and the case.

The amplitude of the triboelectric beam vibration under a respiratory airflow initially remains stable until the velocity of airflow exceeds a critical value. The critical flow speed at which destabilizing the aerodynamic effects is of interest for the design of the device. This speed *U*_C_ could be simply estimated by balancing the bending forces (~*Eh* ^3^ *η*/*L* ^4^) with the aerodynamic forces (~*ρ*_*f*_ *U* ^2^ *η*/*L*):

(10) $$U_{C}\sim {\left(\frac{{\mathrm{Eh}}^{3}}{p_{f}L^{3}}\right)}^{1/2},$$

where *L* is the length of beam, *E* is Young's modulus, *ρ*_*f*_ is the air density, *h* is the beam thickness, and *η* is the tilting angle.^60^ Apparently, the critical velocity is related to the stiffness, geometry of the beam, and air density. Once fluttering occurs, the flutter frequency is determined by the natural frequency of the beam that could be expressed as:

(11) $$f_{i}=\frac{\lambda_{i}^{2}}{2\pi l^{2}}{\left(\frac{\mathrm{EI}}{m}\right)}^{1/2},\;i=1,2,3\ldots ,$$

where *m* is the mass per unit length, *I* is the moment of inertia of the beam, and *λ*_*i*_ is a constant related to the modes of vibration.^61^ This equation applies to both cantilever beam and end‐fixed beam yet *λ*_*i*_ is much different between them. The first three constants of cantilever beam are exemplified, with *λ*_1_ = 4.73, *λ*_2_ = 7.85 and *λ*_3_ = 10.99. The flutter frequency should be equal to its lowest bending mode of natural frequency. Moreover, the complete motion of the beam subjected to respiratory airflow could be given based on the Euler‐Bernoulli equation considering the thin film structure:

(12) $$m\frac{\partial^{2}y}{\partial t^{2}}+\mathrm{EI}\frac{\partial^{2}y}{\partial x^{4}}=l\Delta P,$$

where *l* is the width of beam and *∆P* is the pressure difference on the two sides of beam due to airflow.^62^, ^63^ However, developing an analytical solution to this equation is complicated since the pressure difference is not constant during the vibration. Overall, this type of device usually generates high‐frequency electricity regardless of respiration rate as the frequency is determined by the natural frequency of triboelectric beam.

Another type TENG device is motion triggered which is usually applied to the chest (thoracic respiration) or abdomen (abdominal respiration) to harvest energy from respiration. Specifically, the regular constriction and relaxation of muscle due to respiration deform the device and make periodic displacement, either vertically or laterally (CS and LS modes, respectively), to generate triboelectric output. As exemplified in Figure 1C, a LS‐mode TENG belt is wrapped around the chest. When air is inhaled, the chest expands and stretches the belt to pull the dielectric films apart from each other. When exhales, the chest contracts and the belt relaxes driving the films back to their original places. In response to this type of motion, the electrical output usually synchronizes with the respiration pattern at a low frequency. Details of reported type I and type II devices were compiled and compared in Table 1.

**TABLE 1:** Comparison of typical type I and type II devices harvesting respiration energy

Device type	Working mechanism	Operation locations	Output voltage by respiration (V)	Output current by respiration (μA)	Operation frequency (Hz)	Output power density range	References
Type I	Aerodynamic vibration	Face	0.2‐45	0.5‐18	Up to 600	0.6‐15 W/m^2^	64, 65, 66, 67
Type II	Motion triggering	Chest, abdomen, diaphragm	0.1‐20	<0.3	<2	0.076‐7.584 mW/m^2^	68, 69, 70, 71, 72, 73

### 2.4 Materials design for respiration‐driven TENGs

The triboelectrification effect could be found in almost all materials including polymers, ceramics, metals, and so on. Based on the affinity to electrons, triboelectric series have already been established to serve as guidelines for designing TENGs.^9^, ^74^ Basically, a positively triboelectric material (low affinity to electrons) is usually paired with a negatively triboelectric material (high affinity to electrons) to enable the maximized output. However, regarding to harvesting biomechanical energy from human body, materials with tissue‐compatible properties are more desired. For instance, metal thin film (such as gold, aluminum made by deposition) and low‐modulus polymer film (such as polydimethylsiloxane [PDMS], Kapton, polytetrafluoroethylene [PTFE]) are usually exploited for building flexible/stretchable TENGs.^16^, ^75^, ^76^, ^77^ In the consideration of wearability, TENGs consisting of textile/fabric by weaving triboelectric yarns are developed.^78^, ^79^, ^80^ Those yarns have core‐shell structure with commercial threads (core) and coatings (shell) possessing strong electrostatic effects. Another important material property is the biocompatibility, which must be taken account of when designing the implantable devices.^81^, ^82^ TENGs will receive additional biocompatible encapsulations (mostly PDMS) to avoid inflammatory reaction if they operate inside body.

There are several strategies to enhance the triboelectrification effect in terms of materials selection and engineering. The surface of materials could be modified by physical techniques (such as photolithography and plasma etching) to create nano/micro patterns, which would enhance the surface area and thus the device output.^83^, ^84^ Chemical methods including fluorinated surface, ion injection, sequential infiltration synthesis and molecular‐targeting functionalization could also alter the surface properties and enable high performance.^15^, ^85^ In addition, compositing two or more pure materials such as embedding micro/nanomaterials into polymer matrix would effectively adjust the dielectric constant, and enhance the triboelectric performance of devices as well.^86^, ^87^

In general, clear understanding of the respiration mechanics together with triboelectric principles lays the foundation for the design and engineering of TENG devices that could effectively convert respiration motions into continuous electricity for a variety of biomedical applications.

## 3 BIOMEDICAL APPLICATIONS

The self‐powering capability with other featured properties such as softness, flexibility, biosafety, and biocompatibility opens up many possibilities for TENGs in biomedical engineering. Hitherto, respiration‐driven TENGs have been implemented in a wide range of biomedical applications as a unique and self‐sustainable electrical power/signal source. The applications mainly cover six directions including electrical energy generation, healthcare monitoring, air filtration, gas sensing, electrostimulation, and powering IMDs, which will be discussed in this section.

### 3.1 Electrical energy generation

**FIGURE 2:**
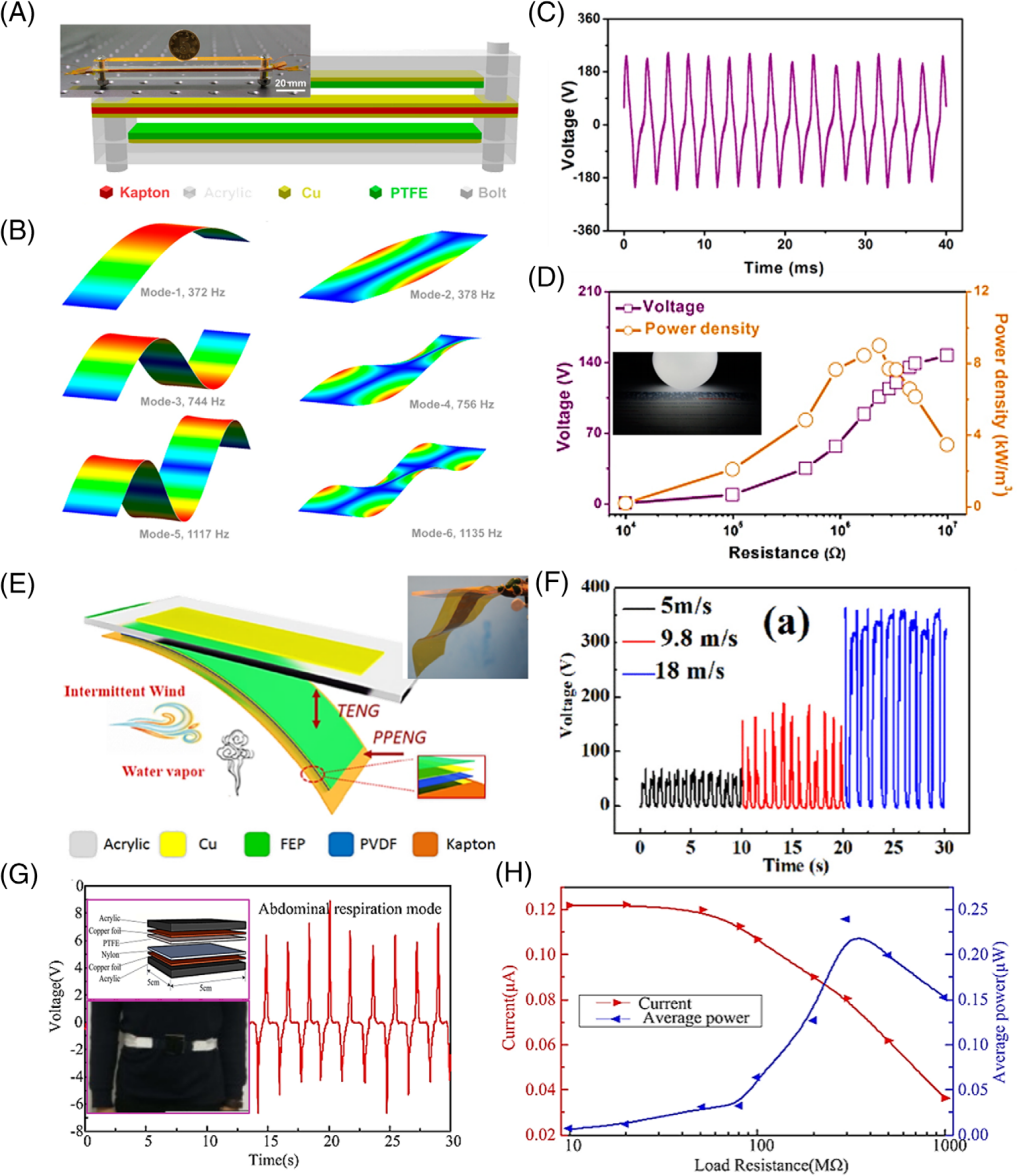
Electrical energy generation by respiration‐driven TENGs. A, Schematic diagram of an aeroelastic vibration TENG. Inset is the digital image of the device. B, Different vibration modes and frequency of the triboelectric beam. C, Output voltage of the TENG under an airflow speed of 15 m/s. D, Voltage and power output as a function of load resistance. Reproduced with permission: Copyright 2015, the American Chemical Society.^64^ E, Schematics of a cantilever TENG. Inset is the digital image of the device. F, Voltage output of the TENG under different airflow speed. Reproduced with permission: Copyright 2018, the American Chemical Society.^65^ G, Voltage output of a CS‐mode TENG when placed on waist. Insets are the schematic and digital image of the TENG device. H, Voltage and power output as a function of load resistance. Reproduced with permission: Copyright 2019, Elsevier.^68^ TENG, triboelectric nanogenerator

The basic function of TENG is to generate electrical energy from mechanical motions. Both types of respiration energy from air flow and chest/abdomen movement have been actively investigated for TENG designs for electricity generation. Aeroelastic vibration devices capable of harvesting respiration energy were developed by multiple investigators. Wang et al reported a device consisting of a triboelectric beam and acrylic frames.^64^ The bottom and top side frames were covered with PTFE and Cu electrodes (Figure 2A), a Kapton film covered by Cu electrodes on both sides was fixed between the acrylic frames. Finite element analysis (FEA) simulation revealed the relationship between airflow and the vibration of the soft beam. Six vibration modes and their corresponding natural frequencies were calculated, with the first‐order and second‐order modes corresponding to bending motion (372 Hz) and twisting motion (378 Hz) that determined the aeroelastic fluttering (twisting frequency higher than bending frequency) (Figure 2B). Under an airflow speed of 15 m/s, this device output a high *V*_OC_ over 180 V at a frequency of 380 Hz (Figure 2C). A maximum power output was estimated to be 9 kW/m^3^ (Figure 2D). Driven by human breathing, output voltage and current can be up to 30 V and 18 μA under a loading resistance of 2.3 MΩ, respectively, resulting in an output power of 0.75 mW. A capacitor of 10 μF could thus be charged from 0 to 1.8 V in only 0.33 second.

In another similar work, Zheng et al demonstrated a cantilever structure with one fixed end.^65^ This device used a Cu‐covered acrylic film as the substrate and electrode‐covered fluorinated ethylene propylene/polyvinylidene fluoride (PVDF) composite film as the cantilever beam (Figure 2E). Under an air flow speed of 5 to 18 m/s, the TENG produced a low‐frequency (1 Hz) voltage at 100‐V level with a maximum of 350 V at 18 m/s (Figure 2F). The significant frequency difference between this cantilever device and the above‐mentioned bridge one is because of the different natural oscillation frequencies based on fixing types and beam materials. The output power of this TENG could be up to 4.74 mW, where an extra power of 184.32 μW was contributed by the piezoelectric PVDF material. Integrated with a face mask, this device could produce sufficient electricity (~45 V and 2.2 μA) to light 31 green LEDs concurrently by harvesting energy from human breath.

In a motion‐triggered device for harvesting respiration energy, a CS mode TENG (nylon and PTFE dielectric films with Cu electrodes) was integrated with a belt through “Z‐shaped” connectors to be applied to the waist.^68^ During respiration, the decreased chest/abdomen circumference retracts connectors causing the full separation of polymer films, whereas expanded chest/abdomen during expiration stretches connectors to render dielectric pairs contact. This device on human volunteers produced a reasonable *V*_OC_ of ~7 V yet a low current around 0.1 μA (Figure 2G). The instantaneous power only reached a peak value of 0.23 μW (Figure 2H). Another similar belt reported by Vasandani et al involved a CS TENG with microstructures. It was able to charge a 1 μF capacitor to 1.261 V in 300 seconds when driven by respiration, corresponding to a power output at the *nW* level.^69^ These power values were significantly lower than those from aeroelastic devices, which were attributed to the coordinated low‐frequency motions between the TENG and the respiration rhythm (~0.7 Hz).

In general, current respiration‐driven TENGs had a power output in the range of 0.1 μW to 1 mW. Although the chest movement contributes to the most mechanical energy, the motion‐triggered devices exhibited significantly lower power output compared to the aeroelastic devices. Considering the large amount of available energy from respiration, both types of devices need to be optimized to largely improve their biomechanical energy utilization rate and energy conversion efficiency. Other useful strategies in TENG designs for harvesting wind flow with high efficiency might be adapted for designing respiration‐driven TENGs with enhanced performance.

### 3.2 Healthcare monitoring

Respiration are closely associated with human health. Electricity output of TENG reflecting respiration characteristics thus may possess a great value for developing self‐powered healthcare monitoring systems with potential applications for respiration behavior recognition, respiration rate and depth monitoring, and respiratory disease detection.^25^, ^26^, ^66^, ^67^, ^68^, ^70^, ^71^, ^72^, ^88^, ^89^, ^90^

**FIGURE 3:**
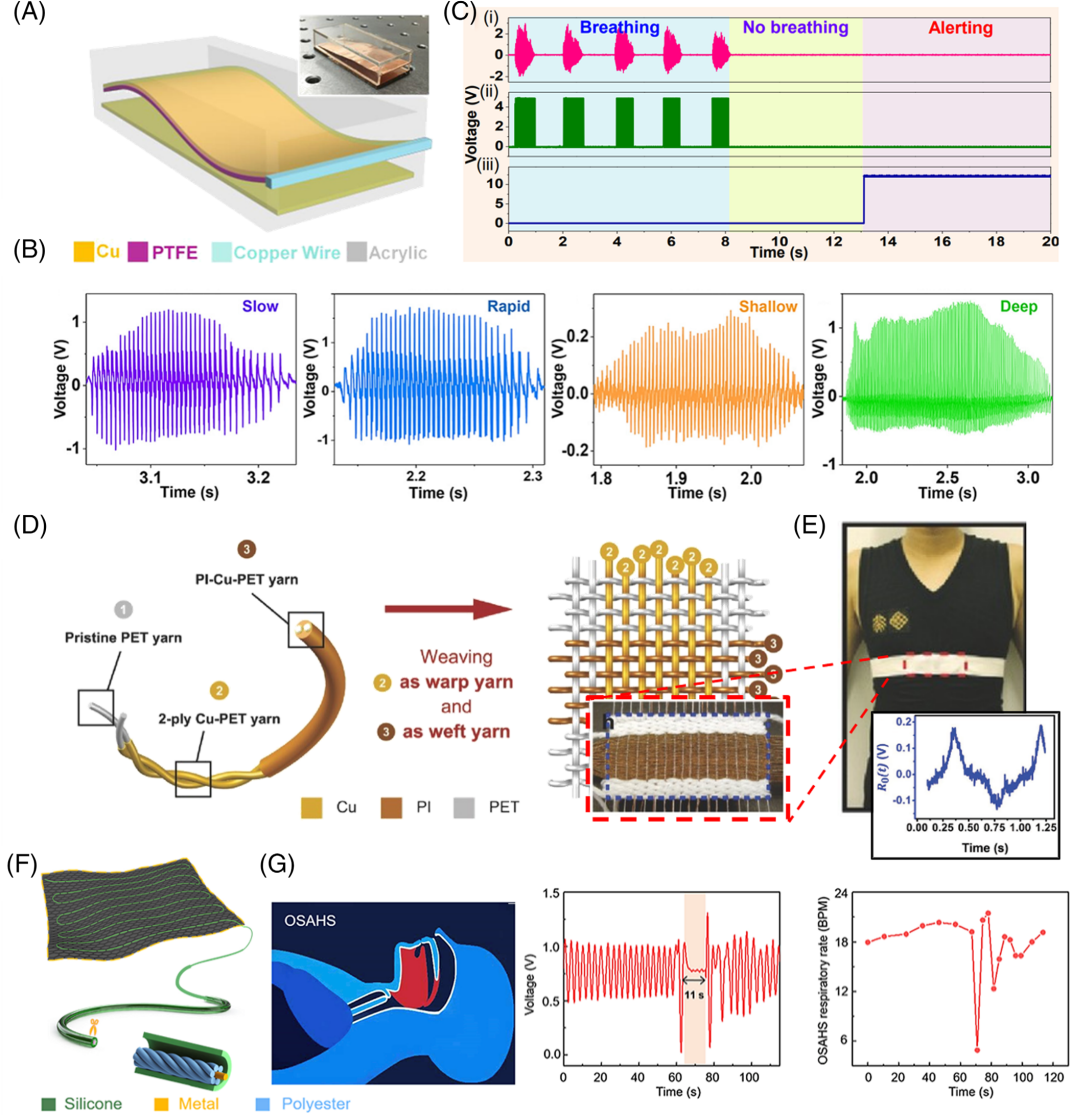
Healthcare monitoring by respiration‐driven TENGs. A, Schematic image of an aeroelastic vibration TENG. Inset is the digital image of the device. B, Real‐time voltage output envelops of slow, rapid, shallow, and deep breathing. C, The signal generated from the TENG (i) converted to square signals by a relay (ii), and the switched signal by a single‐chip microcomputer (iii). Reproduced with permission: Copyright 2018, the American Chemical Society.^66^ D, Schematics of a textile TENG. E, Digital image of the chest strap for respiratory monitoring. Inset is the Raw respiratory signal Reproduced with permission: Copyright 2016, Wiley‐VC.^25^ F, Schematics of the sleep matrix consisting of the textile TENG. G, OSAHS symptom and corresponding detected signals by the sleep matrix. Reproduced with permission: Copyright 2019, Elsevier.^71^ OSAHS, obstructive sleep apnea‐hypopnea syndrome; TENG, triboelectric nanogenerator

Wang et al developed an intelligent wireless TENG system to monitor the airflow during respiration.^66^ The operation of TENG was based on aeroelastic fluttering of a flexible nanostructured PTFE (n‐PTFE) thin film in an acrylic tube (Figure 3A). It was found that the average values of voltage and current rose monotonically from 1.7 to 11.1 V and 0.9 to 10.2 μA, respectively, when the air flow rate increased from 85 to 216 L/min. This monotonic and sensitive response of TENG to airflow rate enabled respiration monitoring. A mask built with the TENG could provide distinguishing electric output features under different respiration patterns (Figure 3B). Specifically, slow breath in sleep induced low‐frequency 1.2 V peaked at the middle of electrical envelop. Rapid breath associated with exercise generated voltage envelop with a larger amplitude up to 1.8 V. Shallow breath in a coma situation exhibited a much weaker voltage of only 0.2 V. Deep breath was featured with a high voltage over 1 V and the longest duration up to 1.3 seconds. Therefore, human respiration behavior and related body status could be directly recognized based on the voltage patterns. Based on this device, an intelligent wireless respiratory monitor and alert system was demonstrated. When the TENG yielded voltage envelopes under normal breathing, active discrete square signal and zero switch signal was provided by the circuit (Figure 3C). Once the breath pattern reached a critical situation (eg, no breath for more than 5 seconds), an alarm would be triggered through a wireless module and a warning massage would be sent to the cell phone. An early intervention could be initiated to help the patient. Instead of alerting at an emergency, Zhang et al also proposed utilizing a similar TENG device to build a human‐machine interaction system to remotely manipulating appliances for disabled people by recognizing their breathing behaviors.^67^

A smart textile TENG (t‐TENG) was developed by Zhao et al to monitor respiration rate and depth.^25^ The t‐TENG was fabricated by direct weaving of Cu‐coated polyethylene terephthalate (Cu‐PET) warp yarns and polyimide (PI)‐coated Cu‐PET (PI‐Cu‐PET) weft yarns on an industrial sample weaving loom (Figure 3D). The energy harvesting fabrics could generate electric output at the yarn crisscross intersections upon any mechanical displacement. A maximum *V*_OC_ of ~5 V was reached. By directly integrating the fabrics into a chest strap, the rate and depth of respiration could be monitored in real‐time (Figure 3E). Expansion and contraction of chest during respiration yielded a *V*_OC_ at the 0.1 V level. Counting the number of voltage peaks during a specific time interval, the respiration rate could be precisely deduced. Furthermore, relationship between tidal volume *V*_T_ and *V*_OC_ was determined to be *V*_T_ ∝ $V_{\mathrm{OC}}^{1/2}$, which allowed evaluation of respiration depth.

Other TENG prototypes monitoring respiratory rate have also been reported. A soft band with a CS‐mode TENG was designed to work as a sheet without direct skin contact. With a high dynamic‐pressure sensitivity of 18.98 V kPa^−1^ within a wide working range of 40 kPa, this device could generate consistent electricity (~0.2 V) when people lying on top of it.^89^ A respiration rate could also be directly obtained from the number of voltage peaks. A smart belt built with a sliding‐mode TENG and a wireless transmission chip was developed by Zhang et al.^72^ The variation of the abdominal cavity circumference induced relative sliding of the triboelectric pair to generate voltage output. The electrical signal could be transmitted through a battery‐powered wireless chip to a mobile phone in real time. Through this device, the respiratory rate was precisely recorded when the person was lying, sitting, standing, and walking.

In addition to respiration monitoring, the direct relationship between breath pattern and voltage output of TENGs could be further used to detect obstructive sleep apnea‐hypopnea syndrome (OSAHS), a respiratory disease due to upper airway obstruction during sleep.^71^ In this work, a conductive yarn composed of polyester and stainless steel was enwrapped with silicone outer sheath to form the triboelectric fiber, with which a single‐layered t‐TENG was fabricated (Figure 3F). The t‐TENG exhibited a high sensitivity of 10.79 mV/Pa at a wide frequency bandwidth (0‐40 Hz). Being placed on a mattress as a bedsheet, this t‐TENG could record the real‐time respiration rate once a patient was lying on top of it. Unlike stable electrical signals from healthy person, the OSAHS patient had distinct blanks in the voltage patterns due to apnea caused by the narrowed airway (Figure 3G). An OSAHS monitoring and intervention system was thus built based on this t‐TENG, which could identify the apnea episodes and provide a timely alarm to the patient for recovering the normal breath.

In general, respiration‐driven TENG devices hold intriguing promises for respiratory healthcare monitoring with a few successful demonstrations. So far, the entire monitoring system usually consists of multiple modules including TENG, signal processor, wireless transmission, signal receiver, and analyzer. These systems are bulky and not fully wearable, raising concerns for convenience and practicality. An all‐in‐one integrated wearable system with miniaturized size is desired. Moreover, the accuracy of long‐term monitoring as well as the stability of TENG system should be further investigated particularly under real application environment.

### 3.3 Air filtration

**FIGURE 4:**
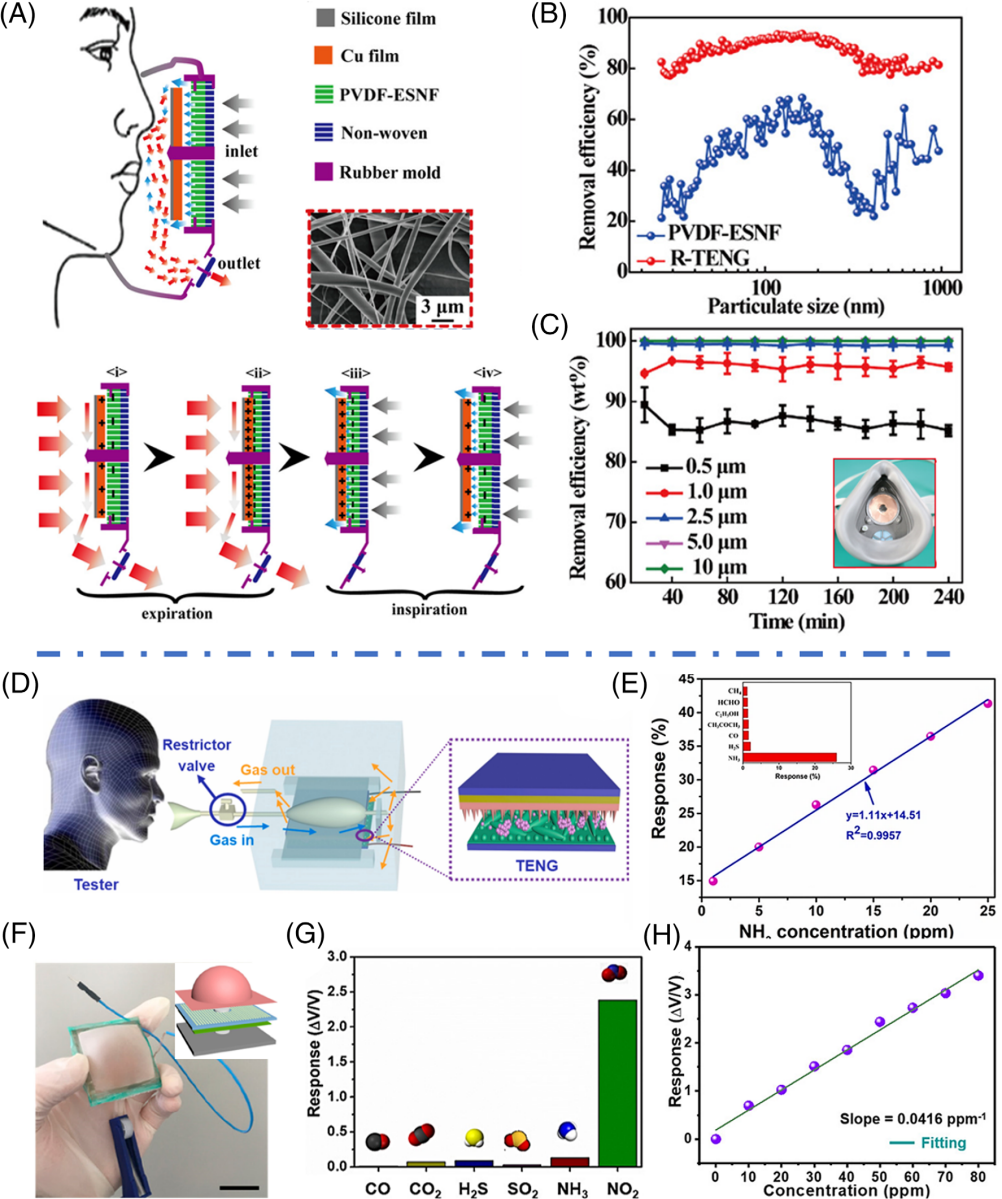
Filtration and gas sensing by respiration‐driven TENGs. A, Schematic of a TENG‐based smart mask filtering particles and its working mechanism. Inset is an SEM image of PVDF‐ESNF. B, Removal efficiencies of PVDF‐ESNF and TENG masks. C, Durability test on removal efficiency of the smart mask integrated with TENG after 30 days. Reproduced with permission: Copyright 2018, the American Chemical Society.^97^ D, Schematic illustration of a respiration‐driven TENG sensor. E, Response‐NH_3_ concentrations fitting curve of the TENG. Inset is the selectivity of TENG to NH_3_ and other interference gases. Reproduced with permission: Copyright 2019, Elsevier.^98^ F, A digital image of a gas‐sensing TENG. Inset is the schematic of the Alveolus‐inspired TENG. G, Selectivity of the TENG to NO_2_ and other interference gases. H, Response‐NO_2_ concentrations fitting curve of the TENG. Reproduced with permission: Copyright 2020, the American Chemical Society.^99^ ESNF, electrospun nanofiber film; PVDF, polyvinylidene fluoride; TENG, triboelectric nanogenerator

Masks are an essential typical of personal protective equipment that filters hazardous particles including viruses from airborne, such as the COVID‐19 virus.^91^, ^92^ Most particulate respirators have layered filters consisting of webs of nanofiber/microfiber to block particles while allow for air to go through. It is based on the mechanisms of gravitational settling, interception, diffusion, and electrostatic attraction.^93^ Since triboelectrification is one of the common electrostatic phenomena, one could either use a TENG to charge the filters or make filters as triboelectric pair to purify the air.^94^, ^95^, ^96^ Liu et al. demonstrated a TENG‐based mask fully driven by respiration.^97^ The inlet of this mask had a Cu film and electrospun PVDF (ESP‐PVDF) on a nonwoven substrate. A positive pressure from expiration pushed the Cu film to make full contact with the ESP‐PVDF layer; while a negative pressure due to inspiration stretched the Cu film back to separate from the ESP‐PVDF. This repeating process could generate and maintain the electrostatic charge on the fiber film (Figure 4A). Therefore, as large particles being removed by physical filtration through the porous structure, small particles were attracted and removed by electrostatic absorption. The TENG‐based mask showed almost the same efficiency as the mask only containing ESP‐PVDF at the initial stage (97.5% particle removal rate). After 240 minutes of continuous work, the TENG‐based mask showed a fairly stable removal efficiency, while this efficiency of non‐TENG masks were significantly reduced. Particularly, for ultrafine particles (25 nm to 1 μm) the TENG‐based mask remained a 77.5% to 93.5% removal efficiency, while the value was dropped to only 32.3% to 68.4% for non‐TENG masks (Figure 4B). Impressively, the TENG‐based mask maintained a removal efficiency over 86.9% for particles below 0.5 μm and over 99.2% for particles over 1 μm for 30 days (Figure 4C), illustrating high removal efficiency and great durability. Although using TENG might significantly enhance the electrostatic adsorption, a disadvantage of this electrostatic mask is that the static charge could fluctuate considerably and would be easily affected by multiple factors including temperature, humidity, and air speed. Particularly, since the exhaled air from respiration contains high humidity, it could largely undermine the electrostatic charge generation capability. To make this intriguing development practical, it is necessary to design and engineer a TENG device that can maintain a high performance with minimal impacts from environmental factors.

### 3.4 Gas sensing

Because the absorption of gas molecules on the tribo‐active material surfaces could change its electrical properties (such as permittivity), the electrical output of TENG would vary upon gas molecule absorption. Therefore, by utilizing gas‐responsive materials as the dielectric materials for charge generation, TENG could be designed for gas sensing without the need of additional power.^98^, ^99^, ^100^, ^101^ Wang et al developed a respiration‐driven TENG system that could detect trace‐level NH_3_ in the exhaled air.^98^ NH_3_ is a biomarker for diagnosing certain diseases (such as ulcers). This system contained an elastic balloon and a CS‐mode TENG consisting of nanostructured PDMS and Ce‐doped ZnO‐polyaniline nanocomposite films (Figure 4D). The balloon was used to store the exhaled air and then to push the air to activate the TENG. Thus, a high sensitive to components in the exhaled air could be achieved. The output voltage monotonously decreased as the concentration of NH_3_ increases. This TENG exhibited a good sensitivity (13.66 ppm^−1^, *R* ^2^ = .9928) when exposed to trace‐level NH_3_ with a concentration from 0.1 to 1 ppm (Figure 4E). An excellent selectivity was also observed to NH_3_ with a significantly higher response value of 0.25 compared to other gases at 0.01‐0.02.

By using WO_3_ as the gas‐responsive material, Su et al made an alveolus‐shaped TENG sensor possessing a high sensibility toward NO_2_, which is the cause of pulmonary edema and respiratory diseases (Figure 4F).^99^ The gas inflation and deflation due to respiration could enable the CS between the latex film and the sensitive WO_3_ film, and generate electricity. A high response of 340.24% and linearity of 0.976 were achieved when this sensor was exposed to 80 ppm of NO_2_ (Figure 4H). In addition, the response toward NO_2_ was over 20 times higher than those from other gases such as CO, H_2_S, SO_2_, NH_3_, and CO_2_ (Figure 4G), indicating an excellent selectivity. Another alcohol analyzer was developed by using TENG as the power source. Instead of directly detecting the adsorbed gas molecules, Wen et al utilized a respiration‐driven sliding‐mode TENG to power a gas sensor for alcohol sensing.^101^ In this design, the solid‐state sensor could be selected and design more independently to TENG material selection. Still powered by respiration energy, this tandem system achieved a high detection gas response of ~34, fast response time of 11 seconds, and recovery time of only 20 seconds.

Compared to other applications, gas sensing of TENG is largely immature. Although a few good gas sensing phenomena of respiration‐driven TENG have been demonstrated, the general performance is still not comparable to regular solid state sensors. One key limitation is the selection of the gas‐sensitive materials, which are typically not strong for triboelectric charge generation. Since it is mostly used on human, several additional issues such as flexibility, biocompatibility, durability, and wearability also need to be investigated and improved.

### 3.5 Electrostimulation

Stimulation and activation of certain cells, organs and systems in human body by low‐dose electricity have long been demonstrated.^102^, ^103^, ^104^, ^105^, ^106^, ^107^ The periodic biphasic pulse‐like electrical signals generated from TENG were also found capable of modulating cell activities, such as accelerating proliferation, adjusting orientation, stimulating motility, and improving differentiation.^32^, ^108^ Therefore, the all‐time accessible respiration energy guarantees a continuous operation of TENG to provide consistent electric pulses for therapeutic electrostimulation.

**FIGURE 5:**
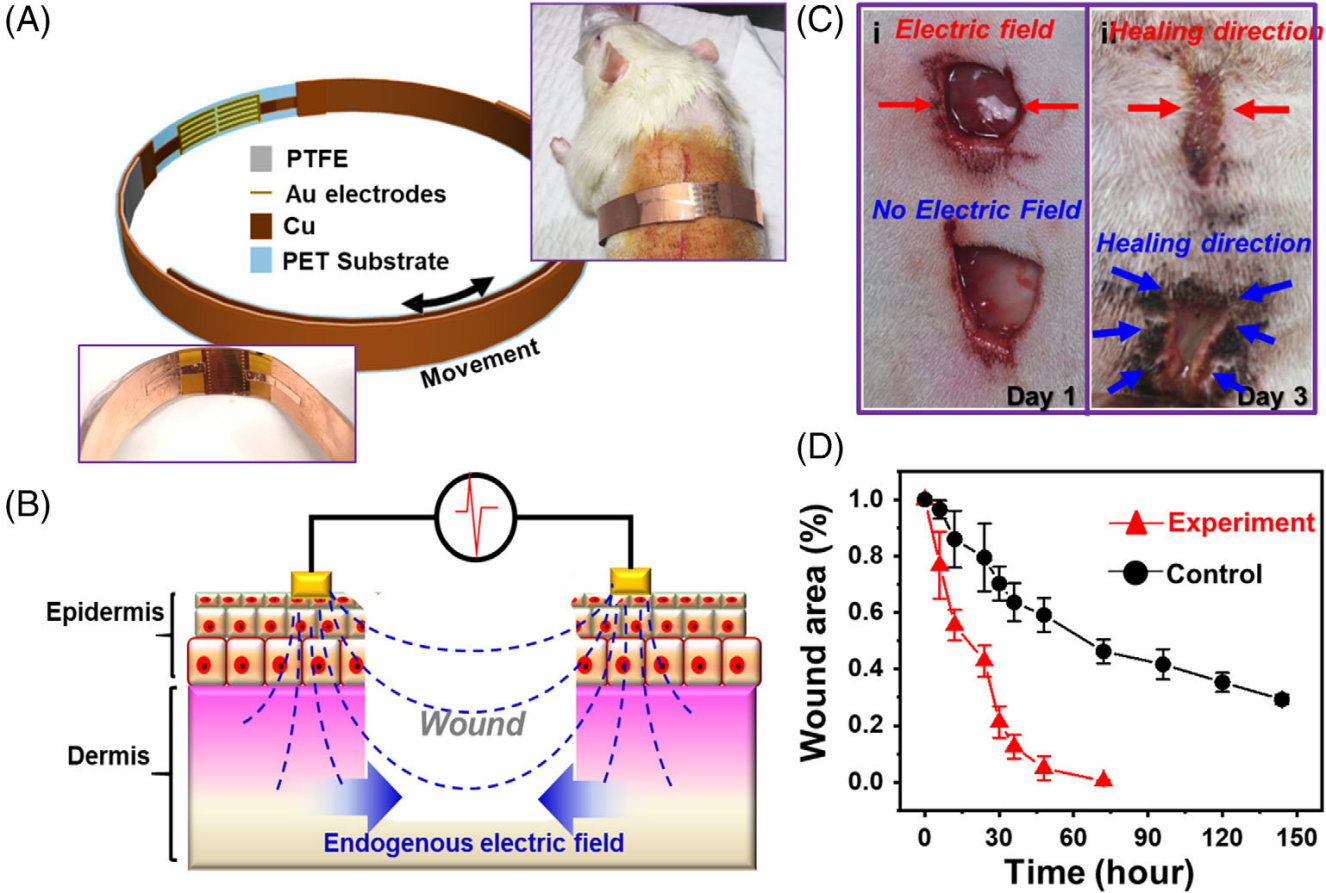
Electrostimulation by respiration‐driven TENGs. A, Schematic of a TENG‐driven electrostimulation device for wound recovery. Insets are a digital image of device (left bottom) and a rat wearing the device (right top), respectively. B, Wound‐healing mechanism under endogenous electric field. C, Digital images of the healing process with and without the TENG electric filed. D, Wound area as a function of time with (Experiment) and without the TENG electric filed (Control). Reproduced with permission: Copyright 2018, the American Chemical Society.^109^ TENG, triboelectric nanogenerator

Long et al fabricated a respiration‐activated electrotherapy bandage consisting of a sliding‐mode TENG and a pair of dressing electrode to accelerate skin wound healing.^109^ The TENG was made by overlapping the metal/PTFE layer with another metal layer on different sides of the PET substrate (Figure 5A). When wrapping the device around the chest area of a Sprague Dawley rat, the expansion and relaxing of chest drove the PTFE layer sliding back and forth and provided a potential of ~1.2 V between the dressing electrodes. The accelerated wound recovery was hypothesized based on the endogenous electric field effect on wound site recovery (Figure 5B). The disruption of transepithelial potential enhanced the endogenous electric field at the wounded area for epithelial cells to initiate directional migration into the dermal wound bed until the skin regeneration process was completed. To demonstrate the accelerated wound healing process, a proximal wound was created on the rat and the two dressing electrodes were aligned over the wound. After 3 days, the wound exposed to an electric field was nearly closed (Figure 5C), whereas the control wound was still largely open. Quantitative study revealed that in the presence of electric fields, the wound reduced rapidly and reached a nearly complete closure (94% ± 4.3%) within the first 48 hours. Without the electric field, the wounded area slowly reduced to 30% within the first 150 hours (Figure 5D). Microscopy study of living fibroblasts showed that the electric field generated by TENG could facilitate the fibroblasts differentiating into myofibroblasts, thereby providing a contraction force for wound closure. Additionally, several important growth factors for wound recovery, such as transforming growth factor beta, epidermal growth factor (EGF), and vascular EGF, all showed enhanced expressions in TENG‐stimulated tissues.

This self‐stimulated electrotherapy for wound healing could have large impacts on a range of related diseases (eg, Raynaud's disease) and may resolve cosmetic concerns, such as chickenpox scars, acne, keloid scarring, or rosacea. One deficiency might be that this motion‐triggered device delivered electricity with a frequency controlled by the respiration rate. Clinical applications of electrotherapy may require an optimal combination of frequency and amplitude to deliver desired treatment. This discrepancy is very challenge to address in current motion‐triggered TENG designs.

### 3.6 Power source for IMDs

Most current IMDs consume energy in the range of μW to mW, while the available energy from respiration could reach up to the Watt level. Owing to the capability of continuous electrical energy generation, respiration‐driven TENG may serve as a unique self‐sustainable electric power supply for IMDs eliminating the bulky and rigid chemical batteries.

**FIGURE 6:**
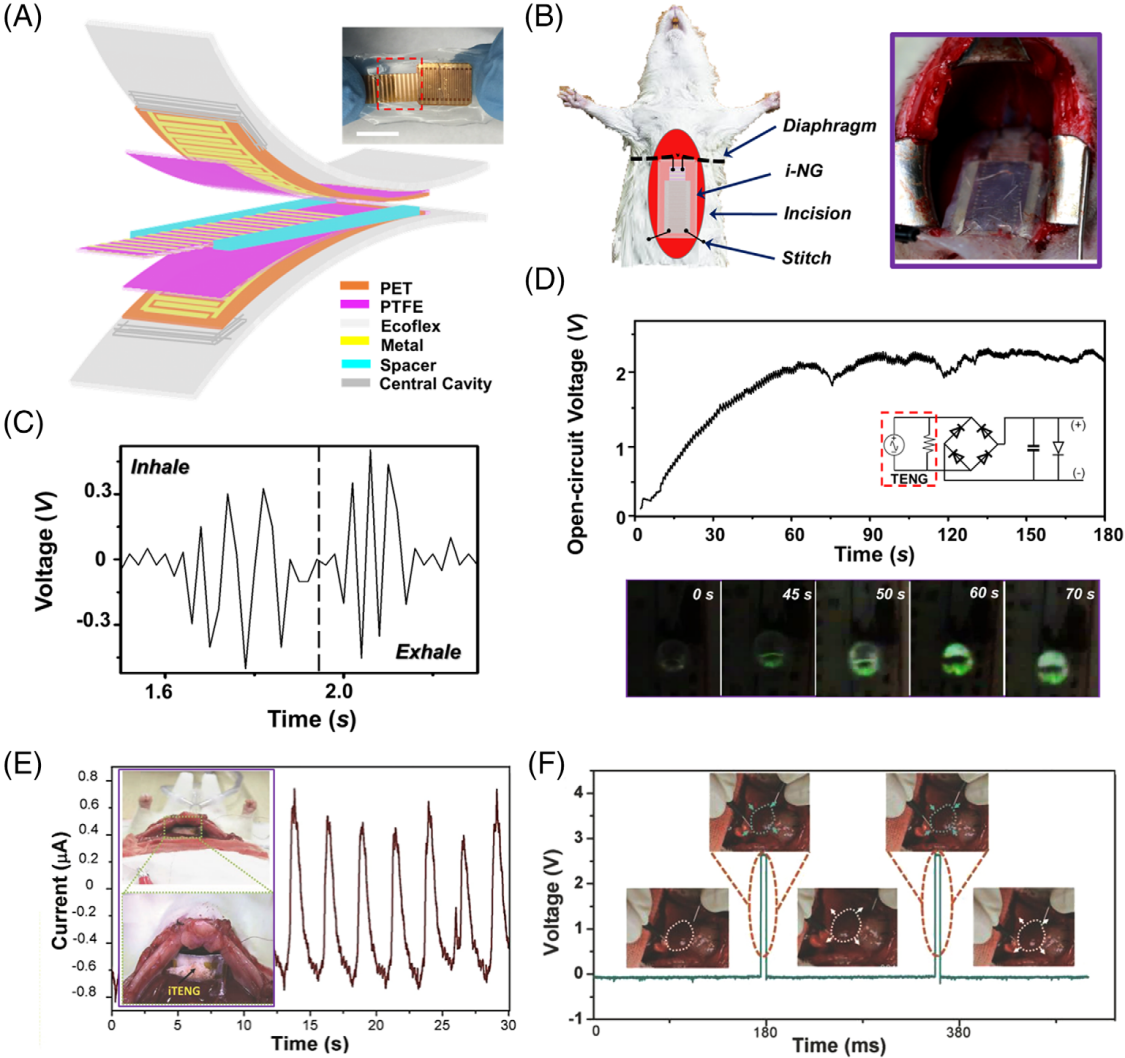
Powering IMDs by respiration‐driven TENGs. A, Schematic of a micrograting TENG for respiration energy harvesting. Inset is a digital image of the TENG. B, Schematic and digital image of the TENG implanted in abdominal cavity driven by diaphragm motion during respiration. C, in vivo voltage outputs measured from the TENG. D, Voltage measured at the LED load as a function of time (upper panel) and a series of images recorded on the LED (bottom panel). Reproduced with permission: Copyright 2018, the American Chemical Society.^73^ E, Current output of a TENG from diaphragmatic movement. Inset is the TENG sutured on diaphragm. F, Stimulation of rat heart by a pacemaker powered by the TENG in vitro. Reproduced with permission: Copyright 2014, Wiley‐VC.^30^ IMDs, implantable medical devices; TENG, triboelectric nanogenerator

Diaphragm moves in a relatively large distance and involuntarily all day around. It is thus considered an ideal in vivo site for biomechanical energy supply. In this regard, Li et al developed an ultrasoft and stretchable implantable sliding mode TENG with higher output current and larger amounts of transported charge compared to CS‐mode TENGs.^73^ This TENG had a micrograting electrode design with a multilayered structure, including two symmetric electrode layers and one mobile layer for electrification (middle layer), packaged by Ecoflex elastomer (Figure 6A). Each electrode layer consisted of Cu/Cr interdigital electrodes (IDEs) deposited on a flexible PET substrate covered by a thin layer of PTFE. The middle mobile layer was comprised of a central PET film sandwiched between two PTFE film where Cu/Cr metal strips were deposited. In a typical micro‐grating configuration, individual fingers of IDEs had a width of 100 to 900 μm (*a*_1_) with a length of 1 cm (*a*_2_) and separated by a gap of 100 μm (*a*_3_). The width (*b*_1_) and gap (*b*_3_) of metal strips in the middle layer were reconfigured accordingly following the relations: *a*_1_ = *b*_1_; and *b*_3_ = 2*a*_3_ + *a*_1_. A central cavity was created inside encapsulation materials to ensure the free move of middle layer when the device was strained. This cavity design also significantly lowered the device's Young's modulus to ~46 kPa, which was comparable to many soft tissues. When implanted in abdomen of a rat by connecting to the diaphragm, this TENG could function as a battery‐free direct current micropower supply (Figure 6B). During normal breath, the up and down movement of diaphragm periodically stretched and relaxed the device, leading relative sliding of the center layers to produce groups of electric spikes (~0.8 V measured at 1 MΩ) as shown in Figure 6C. When connected through a set of rectifier and capacitor, a LED could be continuously and stably lighted up by the anesthetized rat at a breath rate of 45/min after experiencing a short charging period (50‐60 seconds) (Figure 6D). This result successfully demonstrated the feasibility of continuously powering a small electronic device solely by harvesting biomechanical energy.

As one of the most important and broadly used IMDs, cardiac pacemaker consumes electrical energy of 10‐15 μW, which is within the range of typical TENG outputs. Zheng et al. demonstrated the promises of powering a pacemaker by a respiration‐driven TENG.^30^ Such a self‐powered pacemaker prototype was built by integrated a CS‐mode TENG with a pacemaker through a rectifying bridge and a capacitor. The TENG was fabricated from a microstructured PDMS film and an Al foil. The TENG device was either implanted under the chest skin or fixed in between the diaphragm and the liver. When the rat was in anesthesia, the device positioned on chest produced an AC electricity of ~±3 V and ~±0.1 μA at a fast respiration rate of ~1 Hz. The one placed under diaphragm output higher current up to 0.6 μA (Figure 6E) at a lower respiration rate (0.4 Hz). Through the full‐wave rectifying bridge, a 10 μF capacitor was charged from 2 to 3 V within 275 minutes by a TENG in vitro (equivalent to 13 750 breathing cycles if implanted under chest). This amount of electrical energy was used by the pacemaker to generate heart stimulation pulses at various frequencies from 2 to 5 Hz to regulate the heart rates of the rat (Figure 6F). In general, the respiration‐driven TENG holds great promises as a self‐sustainable power source for IMDs. However, the few preliminary studies are still very far away from clinical applications. Many more fundamental developments including packaging, long‐term impact to respiration, stability of electricity output, and power management and regulation need to be investigated to support future clinical studies.

### 3.7 Comparison with other technologies

Other technologies such as PENG^110^, ^111^, ^112^, ^113^, ^114^ and electromagnetic generator (EMG)^115^, ^116^, ^117^, ^118^, ^119^ have the capability of harvesting respiration energy and realize self‐powered biomedical system as well. However, the TENGs still stands out with unique merits. Table 2 compiles detailed information of each technology. Apparently, TENG has outperformed PENG in terms of maximal power and energy conversion efficiency, although they have quite similar properties in other aspects (such as weight and size). The concurrent TENG driven by human respiration could achieve 0.75 mW output, which was over 10 times higher than that of PENG. TENGs possess huge advantages over EMGs when the device volume and weight are critical, because they have close maximal power. The new generation of biomedical devices has the trend toward miniaturization and light weigh where the EMGs could hardly be competent.

**TABLE 2:** Comparison of respiration driven triboelectric nanogenerator (TENG) and other energy technologies

	Respiration‐driven TENG	Respiration‐driven PENG	Respiration‐driven EMG	Flexible/stretchable battery
Mechanism	Triboelectrification and electrostatic induction	Piezoelectricity and electrostatic induction	Electromagnetic induction	Electrochemical reactions
Delivered electricity	Voltage/current spikes/envelopes	Voltage/current spikes/envelopes	Voltage/current spikes/envelopes	Constant electricity over time
Device weight (g)	~1–10	~1‐10	>100	~10‐100
Output power	0.23 μW to 0.75 mW	4‐63.63 μW	3.1 μW to 1 mW	54.3‐1200 W h kg^−1^
Operating voltage	0.1‐45 V (human breathing)	75 mV to 5 V (human breathing)	20 mV to 1 V (human breathing)	0.3‐4.2 V
Lifetime	Stable output over 10 000 cycles	Stable output over 3000 cycles	Extremely long	Stable output for 40‐2000 cycles (without strain)
Applications	Energy harvester, sensor, smart mask, electroceuticals, power source	Energy harvester, sensor	Energy harvester, sensor	Power source for wearable and implantable devices
References	25, 30, 41, 64, 65, 66, 67, 68, 69, 70, 71, 72, 73, 88, 89, 90, 97, 98, 99, 100, 101, 109, 120, 121, 122	110, 111, 112, 113, 114	115, 116, 117, 118, 119	123, 124, 125, 126, 127, 128

While the majority of biomedical devices are still powered by batteries with rigid encapsulation, the batteries could contribute up to 90% weight or volume of the entire device.^48^, ^129^ Despite the emergence of flexible/stretchable batteries opening up possibilities for wearable and implantable bioelectronics,^123^, ^124^, ^125^, ^126^, ^127^, ^128^ other potential issues including electrode delamination during deformation and leakage of toxic electrolyte still raise concerns for its practical applications.^130^, ^131^ Moreover, taking account of IMDs, the replacement of or recharging the batteries requires substantial surgical or technical efforts, introducing additional suffering and complexity (infections and inflammations) to the patients. On the contrary, smaller, lighter, safer, and more effective and durable TENGs with constant output exhibit great promise in broad biomedical applications.

## 4 CONCLUSIONS AND PROSPECTIVE

**FIGURE 7:**
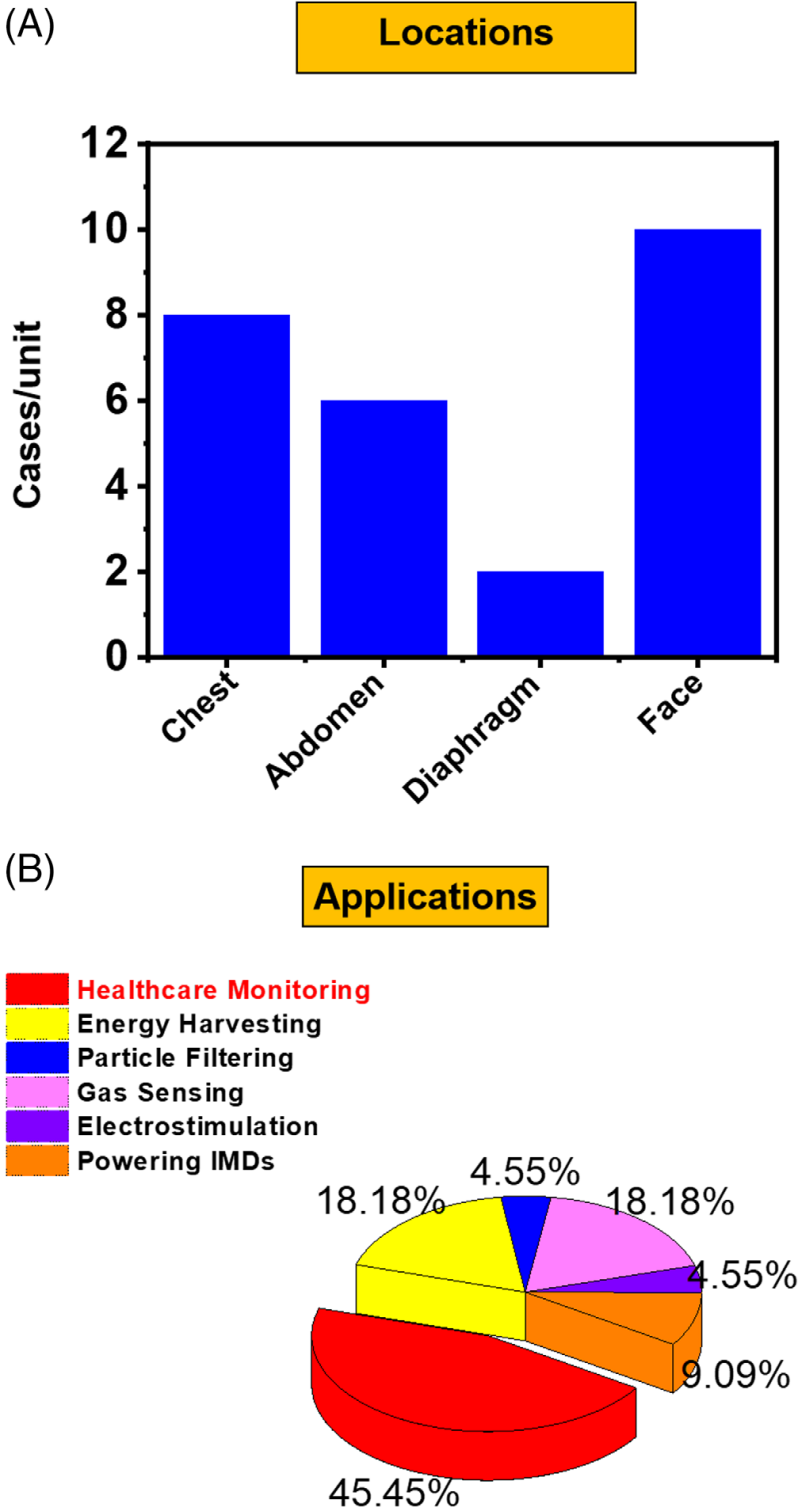
Summary of respiration‐driven TENGs. A, Diagram showing the number of reports where a TENG was activated at different locations during respiration. B, Pie chart showing the percentage of different application demonstrations of respiration‐driven TENGs. TENG, triboelectric nanogenerator

This article provides an overview of respiration‐driven TENG from both fundamental and application perspectives. It is well recognized by the community that the energy from respiration is sufficient and accessible, which can consistently fuel TENG devices to achieve versatile biomedical applications, such as electrical energy generation, healthcare monitoring, air filtration, gas sensing, electrostimulation, and powering IMDs. As summarized in Figure 7A, due to the best accessibility, 70% respiration‐driven TENGs were designed for chest and mouth/nose operation. Among all application demonstrations, healthcare monitoring is the mostly studied application (>45%) primarily because that respiration is an important physiological indication closely associated with multiple health problems (Figure 7B). Despite the exciting early‐stage demonstrations of respiration‐driven TENGs, there exist a few critical challenges that need to be addressed to fulfill their promising role in practical applications.

1. *Energy utilization and conversion efficiency*. Although theoretical calculations and in‐lab studies have shown a high power density (up to kW/m^3^) and high energy conversion efficiency (~80%) of TENG, respiration‐driven TENGs only output an electrical power in the range of 0.1 μW to 1 mW. Given the available respiration energy of an average adult is ~0.1 to 1 W, the so far achieved energy conversion efficiency was less than 1%. The most likely reason was related to the low mechanical energy utilization. Although all TENGs utilized flexible polymers, the device modulus was still orders of magnitudes higher than that of body tissues. Designing TENGs with tissue‐comparable mechanical properties may maximize the mechanical displacement in response to respiration actions and thus reduce the mechanical energy loss.
2. *Long‐term device accuracy and stability*. Triboelectrification as a typical electrostatic phenomenon could be easily affected by the ambient environment, particularly temperature and humidity. Though researchers have claimed that some specific designs of TENG could work under a wide range of humidity and temperature, the long‐term effect of these elements on the performance of respiration‐driven TENG (such as accuracy of monitoring and sensing) has not been systematically studied. Appropriate packaging strategies without affecting device performance should be developed to isolate the functional component of a TENG from the environment. Meanwhile, the durability of the TENG due to mechanical and chemical damage during friction should be considered simultaneously.
3. *Interference with other body movement*. TENG can pick up movements from all sensible body actions, which may result in interference with the electrical signals from respiration and lead to wrong conclusions. For a respirator monitoring TENG placed on chest, large movements from arms could also induce chest motions and make TENG to respond. This additional electrical signal would interfere the electrical signal for respiration rate or depth monitoring. How to differentiate the target signal from other interferences is critical for the achieving accurate sensing and monitoring. A potential solution might rely on advanced algorithms to filter interference and extract targeted data.
4. *All‐in‐one self‐powered system*. At last but not least, while TENG is a small and flexible membrane‐like device, the entire biomedical electronic system may contain many other modules for signal processing, transmission, receiving, and analyzing. Operation of multiple modules still require extra power supplies, and thus diminishes the meaningfulness of achieving a self‐powered biomedical application. How to design an all‐in‐one miniaturized system that is fully integrated with and powered by a TENG device is of great significance as well as a great engineering challenge. This challenge is also beyond respiration‐driven TENGs and is applicable to all other implantable energy harvesting devices for powering IMDs.

Above critical challenges need short or long‐term dedicated research efforts from multiple disciplines, including material science, mechanical engineering, electrical engineering, and of course biomedical engineering. Nevertheless, owing to its unique advantages, respiration‐driven TENG technology will likely play a significant role in the next generation power sources for a wide range of biomedical applications.
